# Digital data of quality control strains under general deposit at Microbial Culture Collection (MCC), NCCS, Pune, India: A bioinformatics approach

**DOI:** 10.1016/j.dib.2016.04.048

**Published:** 2016-04-26

**Authors:** Bhagwan N. Rekadwad, Chandrahasya N. Khobragade

**Affiliations:** School of Life Sciences, Swami Ramanand Teerth Marathwada University, Nanded, Maharashtra 431606, India

**Keywords:** Chaose Game Representation, GC content, Microbial Culture Collection, QR codes, Standard type strains

## Abstract

A total of 13 short DNA sequences of quality control strains (MCC 2052, MCC 2077, MCC 2078, MCC 2080, MCC 2309, MCC 2322, MCC 2408, MCC 2409, MCC 2412, MCC 2413, MCC 2415, MCC 2483 and MCC 2515) were retrieved from NCBI BioSample database and generated quick response (QR) codes for sequences. 16S rRNA was used for creation of Chaose Game representation (CGR), Chaose Game Representation of Frequencies (FCGR) and measurement of GC percentage. Digital data in the form of QR codes, CGR, FCGR and GC plot would be useful for identification, visual comparison and evaluation of newly isolated strains with quality control strains. The digital data of QR codes, CGR, FCGR and GC content all the quality control strains are made available to users through this paper. This generated digital data helps to evaluate and compare newly isolated strains, less laborious and avoid misinterpretation of newly isolated species.

## **Specifications Table**

**Table:** 

Subject area	*Microbiology*
More specific subject area	*Bioinformatics*
Type of data	*Table, figure and graphs*
How data was acquired	*Through NCBI BioSample database*
Data format	*Analyzed*
Experimental factors	*Bioinformatics tools were used for creation of digital information*
Experimental features	*Analysis and digitization of DNA sequences were carried out using bioinformatics approach*
Data source location	Microbial Culture Collection, National Centre for Cell Sciences, Pune, Maharashtra, India (18° 32′ 14.00″ N; 73° 47′ 29.79″ E)
Data accessibility	Data available within this paper.

## **Value of the data**

• Digital information generated provides most comprehensive and analysed data on quality control strains present under general deposit facility in Microbial Culture Collection, National Centre for Cell Sciences, Pune, Maharashtra, India.
• Data would be valuable for identification, evaluation and comparison of other species with quality control strains.
• Data would be valuable for analysis and interpretation correlation between GC content and thermophilic nature of bacteria.
• These experiments with quality control strains were carried out first time by us and made available to users.

## 1 Data

**Fig. 1:**
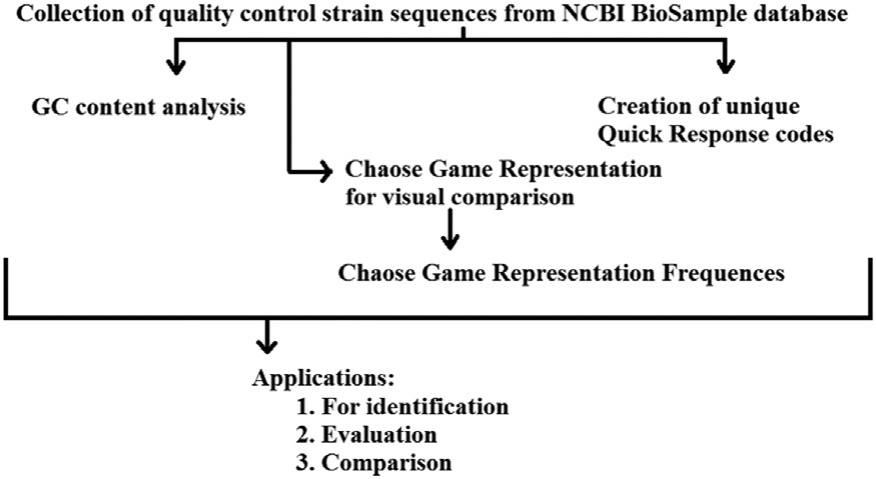
Flow diagram for digitisation of quality control strains and their application.

**Fig. 2:**
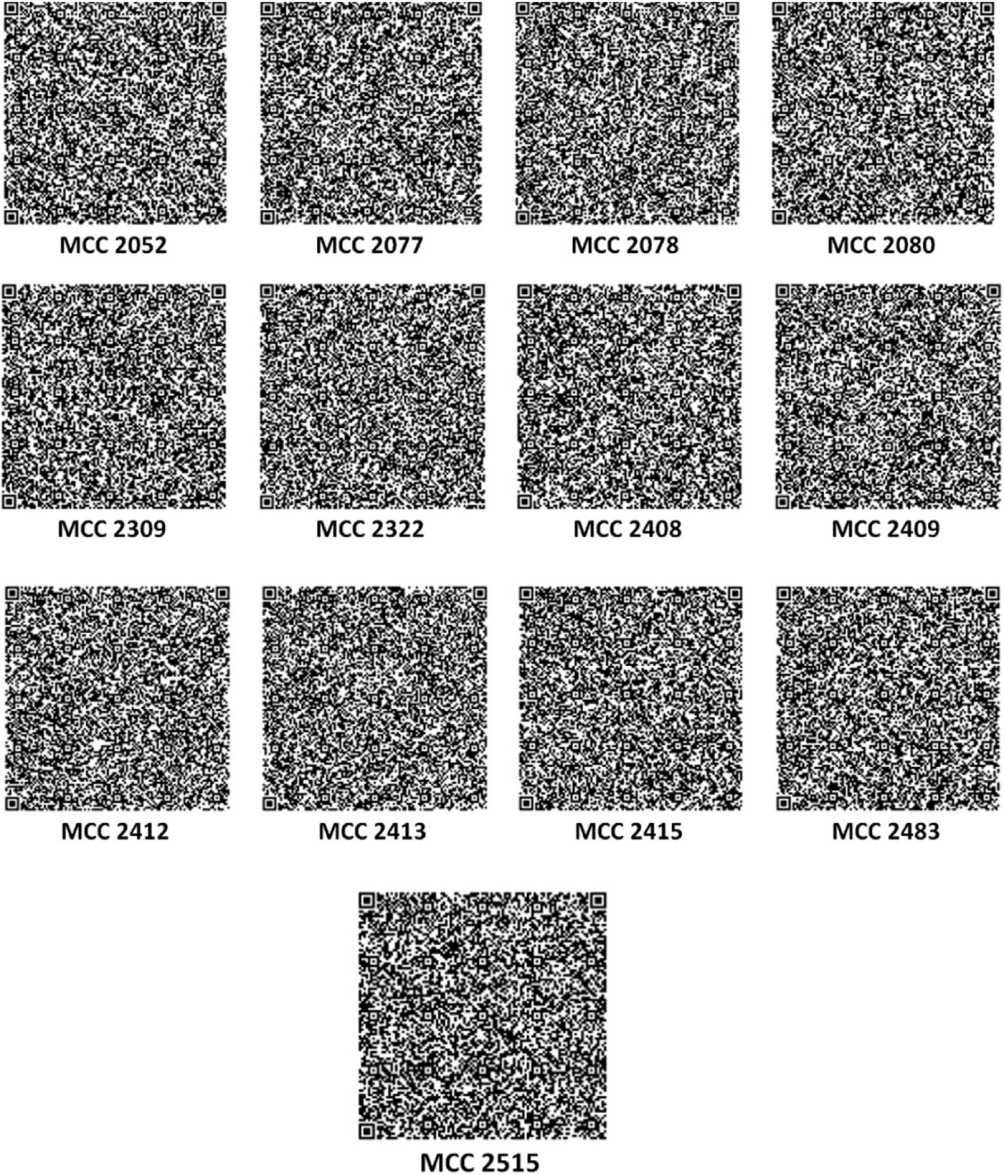
QR codes of quality control strains.

**Fig. 3:**
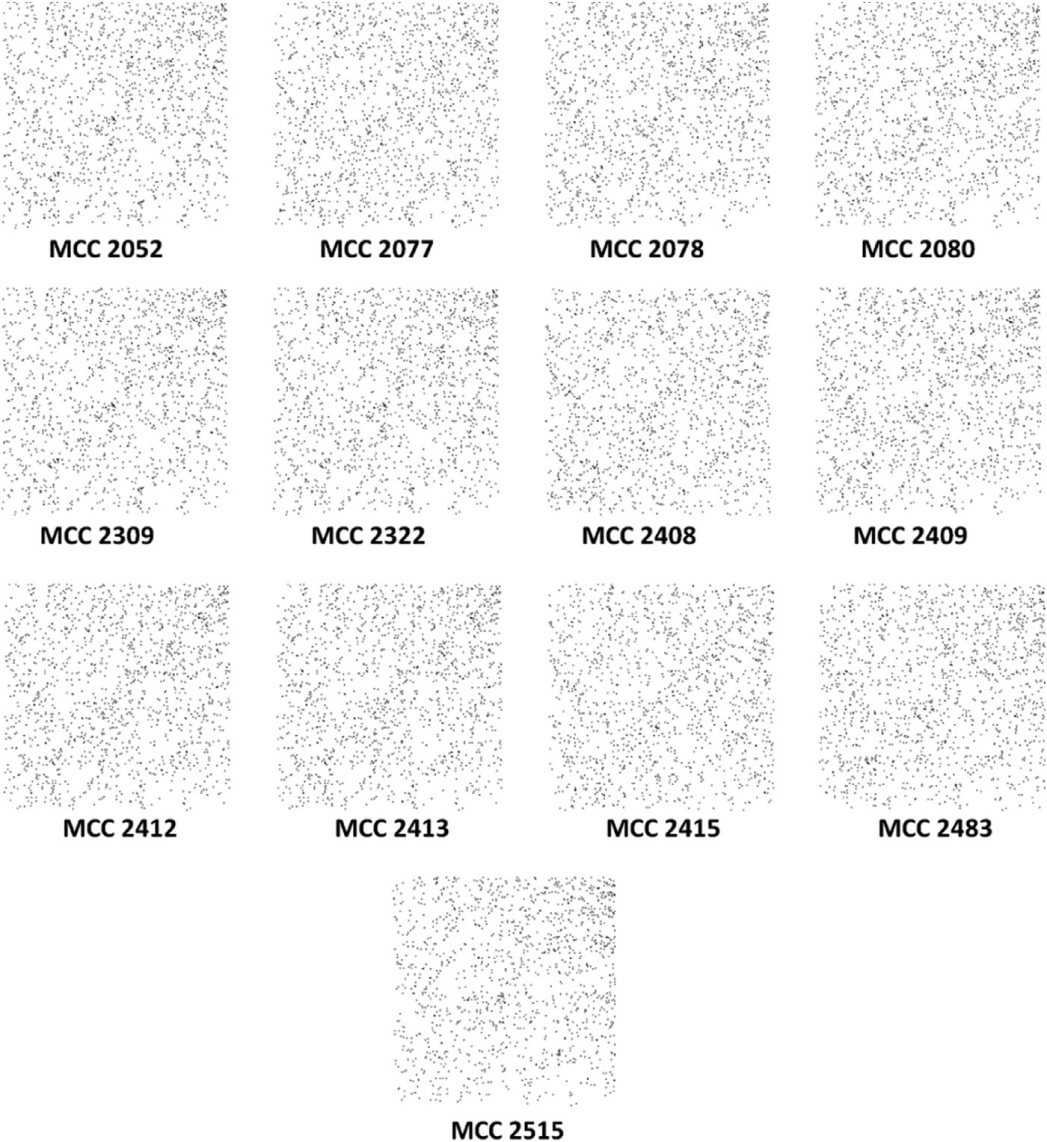
Chaose Game Representation of quality control strains.

**Fig. 4:**
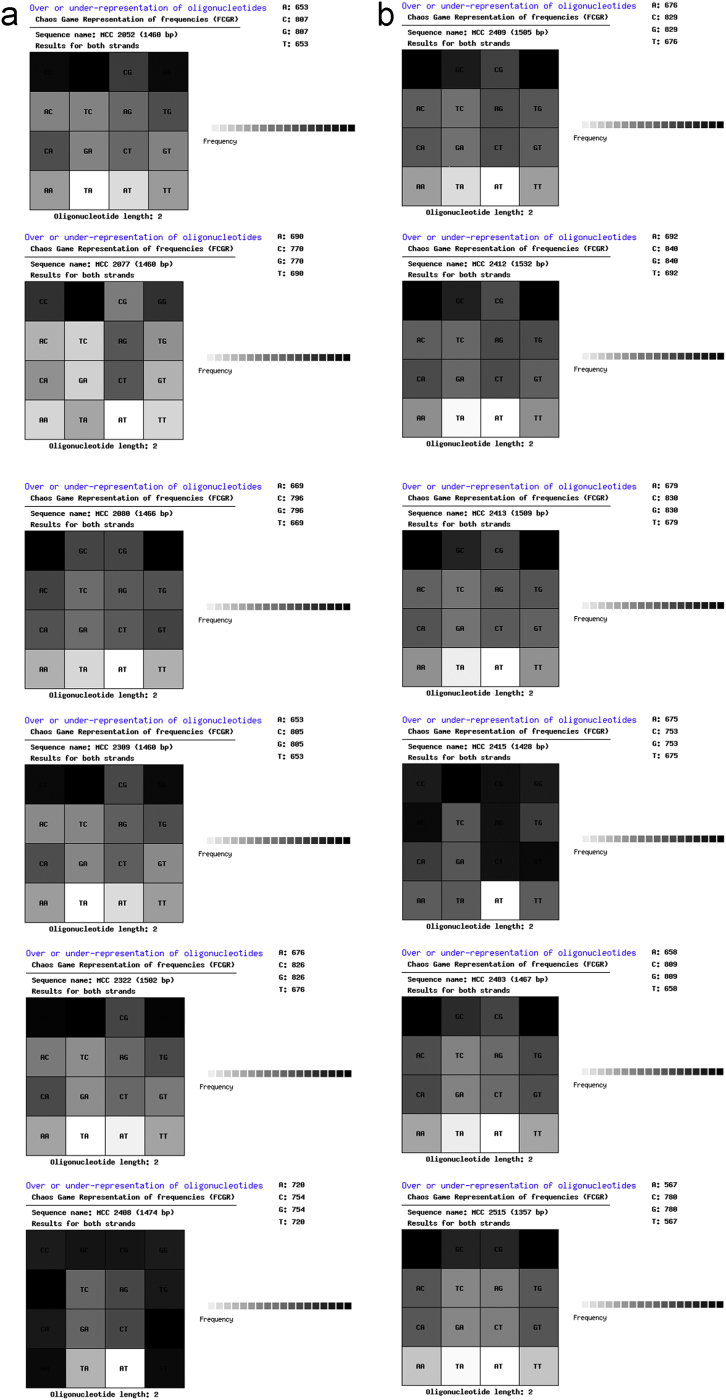
Chaose Game Representation of Frequencies: quality control strains.

**Fig. 5:**
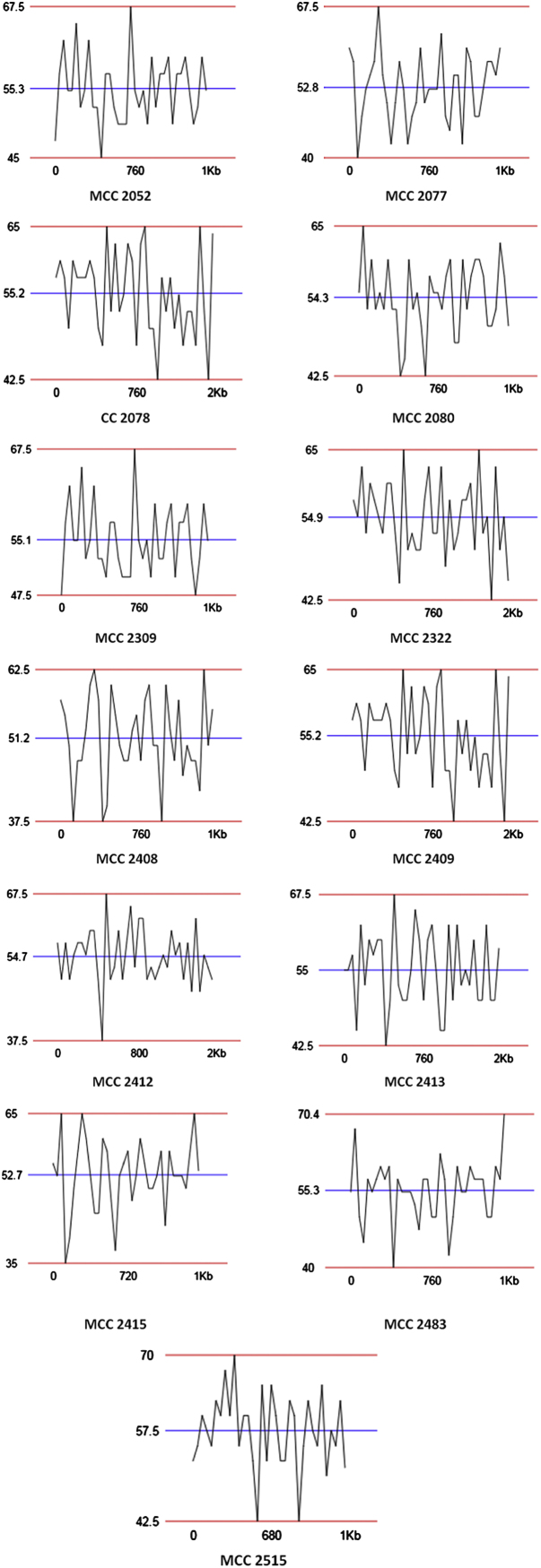
GC percentage in short DNA sequences quality control strains under general deposit at MCC, NCCS, Pune, Maharashtra, India.

A total of 13 short DNA sequences of quality control strains (MCC 2052, MCC 2077, MCC 2078, MCC 2080, MCC 2309, MCC 2322, MCC 2408, MCC 2409, MCC 2412, MCC 2413, MCC 2415, MCC 2483 and MCC 2515) were obtained through NCBI׳s BioSample database. QR codes for these species were generated through DNABarID tool. Chaose Game Representation (CGR) and CGR of Frequencies were graphically represented using BioPHP tool [1]. Data on GC content in percentage were generated using ENDMEMO GC calculating tool (Table 1 and Fig. 1, Fig. 2, Fig. 3, Fig. 4, Fig. 5) [2]. See also *NCBI repository* http://www.ncbi.nlm.nih.gov/nuccore and MCC – list of bacteria under general deposit http://www.nccs.res.in/mcc/Bacteria.html.

## 2 Experimental design, materials and methods

Short DNA sequences in FASTA format were retrieved from NCBI BioSample database. The data in the form of QR codes, CGR, FCGR and GC plot was generated using DNABarID, BioPHP and ENDMEMO GC calculating and plotting tools respectively. DNABarID tool was used for creation of unique QR codes. BioPHP tool was used for graphical representation of nucleotide in quality control strains. ENDMEMO tool was used for calculation of GC content and plotting GC distribution graph.

## 3 Interpretation of generated digital data

The generated QR codes, CGR, FCGR and GC plot using 16 S rRNA sequences of quality control strains would be useful for identification, evaluation and comparison of newly isolated species. The data displayed in QR codes is exactly similar to the data available on NCBI web portal. The number of dots appeared in a CGR image is directly proportional to number of base pairs in a field. Like CGR, FCGR images also represent the concentration of base pairs in a quantitative manner. FCGR comprises twelve squares which mimics Ramanujan׳s Magic Square but different than it. Dark colour of square indicates that higher frequency if base in 16 S rRNA. Frequency tape given in each figure is helpful to understand pattern of distribution of base pairs in DNA. Darkness of the square is directly proportional to the number of base pairs in the square. GC plot shows maximum (upper; red), average (middle; blue) and minimum (lower; red) GC percent lines

I) Upper red line indicate maximum % of GC.
II) Middle blue line indicate average % of GC.
III) Lower red line indicate minimum % of GC in short DNA sequence.

## Figures and Tables

**Table 1:** Details of digitised short DNA sequences of quality control strains under general deposit at MCC, NCCS, Pune, Maharashtra, India.

**MCC accession no.**	**Name**	**NCBI BioSample ID (Accession no.)**	**Maximum GC content**	**Average GC content**
2052	*A. hydrophila* (ATCC 7966 T)	X74677	67.5	55.3
2077	*A. calcoaceticus*	X81661	67.5	52.8
2078	*Citrobacter freundii* 16 S rRNA gene (strain DSM 30039)	AJ233408	65	52.2
2080	*Pseudomonas aeruginosa* strain ATCC 27853	AY268175	65	54.3
2309	*A. caviae* (ATCC 15468 T)	X74674	67.5	55.1
2322	*Aeromonas caviae*	X60409	65	54.9
2408	*Staphylococcus aureus* subsp*. aureus*	AB594753	62.5	51.2
2409	*Citrobacter freundii*	AJ233408	65	55.2
2412	*Escherichia coli* (strain ATCC 25922)	DQ360844	67.5	54.7
2413	*Escherichia coli*	AM980865	67.5	55
2415	*Streptococcus pneumoniae*	AY281082	65	52.7
2483	*Stenotrophomonas maltophilia*	AB008509	70.4	55.3
2515	*M. phlei*	M29566	70	57.5
