# Supplementary figures and images for: Digital data of quality control strains under general deposit at Microbial Culture Collection (MCC), NCCS, Pune, India: A bioinformatics approach
Source: Data Brief. 2016 Apr 26;7:1524–30. doi: 10.1016/j.dib.2016.04.048 (PMC4865665; doi:10.1016/j.dib.2016.04.048)

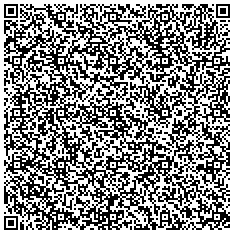

Supplement: Supplementary file 2 — Supplementary material [file mmc2.zip › QR2052.jpg]

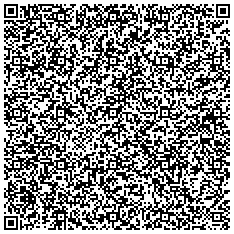

Supplement: Supplementary file 2 — Supplementary material [file mmc2.zip › QR2077.jpg]

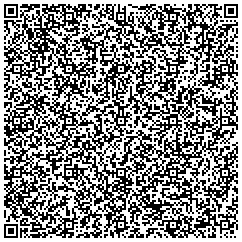

Supplement: Supplementary file 2 — Supplementary material [file mmc2.zip › QR2078.jpg]

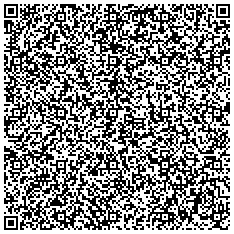

Supplement: Supplementary file 2 — Supplementary material [file mmc2.zip › QR2080.jpg]

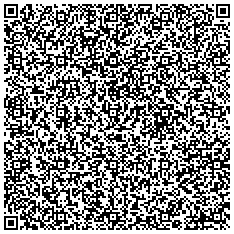

Supplement: Supplementary file 2 — Supplementary material [file mmc2.zip › QR2309.jpg]

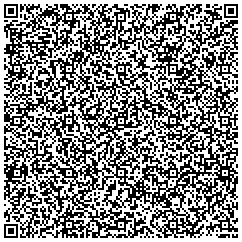

Supplement: Supplementary file 2 — Supplementary material [file mmc2.zip › QR2322.jpg]

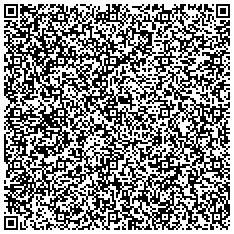

Supplement: Supplementary file 2 — Supplementary material [file mmc2.zip › QR2408.jpg]

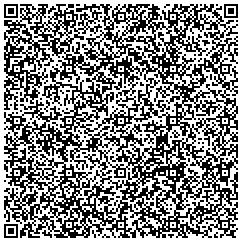

Supplement: Supplementary file 2 — Supplementary material [file mmc2.zip › QR2409.jpg]

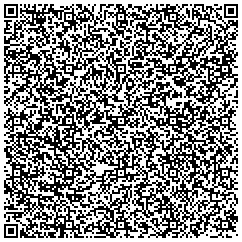

Supplement: Supplementary file 2 — Supplementary material [file mmc2.zip › QR2412.jpg]

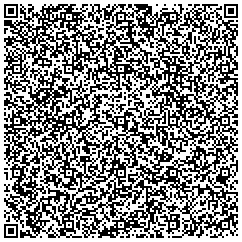

Supplement: Supplementary file 2 — Supplementary material [file mmc2.zip › QR2413.jpg]

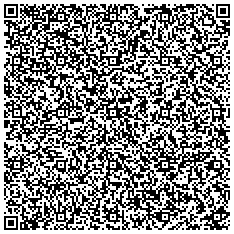

Supplement: Supplementary file 2 — Supplementary material [file mmc2.zip › QR2415.jpg]

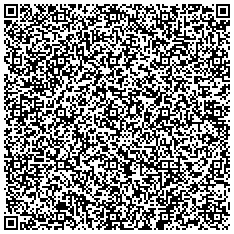

Supplement: Supplementary file 2 — Supplementary material [file mmc2.zip › QR2483.jpg]

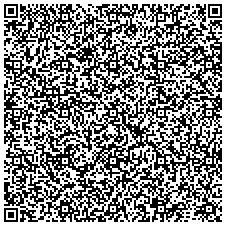

Supplement: Supplementary file 2 — Supplementary material [file mmc2.zip › QR2515.jpg]

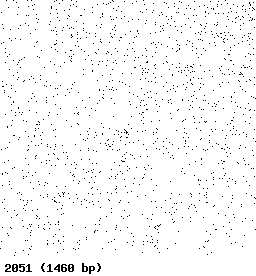

Supplement: Supplementary file 3 — Supplementary material [file mmc3.zip › CGR 2052.png]

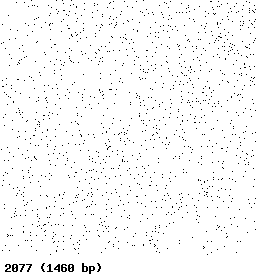

Supplement: Supplementary file 3 — Supplementary material [file mmc3.zip › CGR 2077.png]

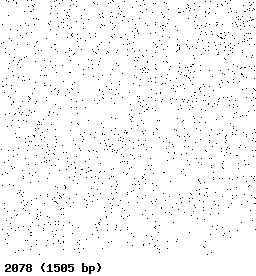

Supplement: Supplementary file 3 — Supplementary material [file mmc3.zip › CGR 2078.png]

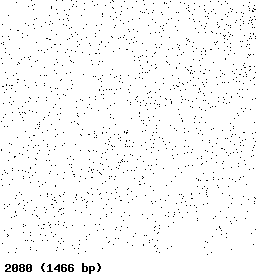

Supplement: Supplementary file 3 — Supplementary material [file mmc3.zip › CGR 2080.png]

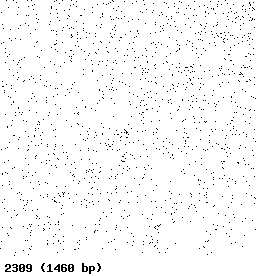

Supplement: Supplementary file 3 — Supplementary material [file mmc3.zip › CGR 2309.png]

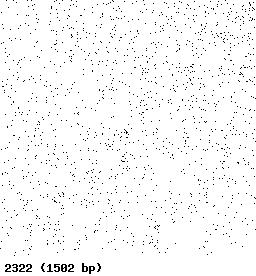

Supplement: Supplementary file 3 — Supplementary material [file mmc3.zip › CGR 2322.png]

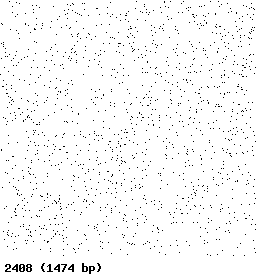

Supplement: Supplementary file 3 — Supplementary material [file mmc3.zip › CGR 2408.png]

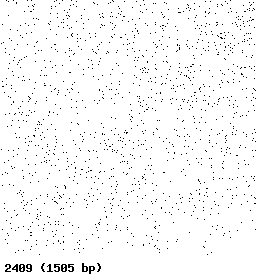

Supplement: Supplementary file 3 — Supplementary material [file mmc3.zip › CGR 2409.png]

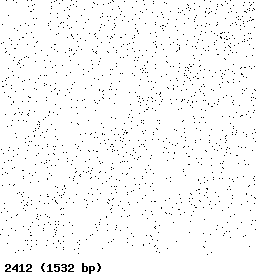

Supplement: Supplementary file 3 — Supplementary material [file mmc3.zip › CGR 2412.png]

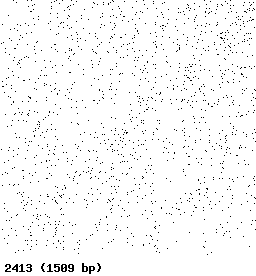

Supplement: Supplementary file 3 — Supplementary material [file mmc3.zip › CGR 2413.png]

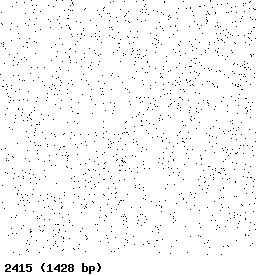

Supplement: Supplementary file 3 — Supplementary material [file mmc3.zip › CGR 2415.png]

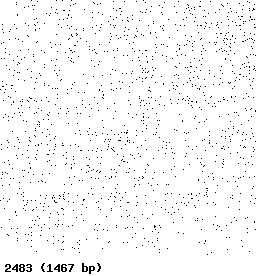

Supplement: Supplementary file 3 — Supplementary material [file mmc3.zip › CGR 2483.png]

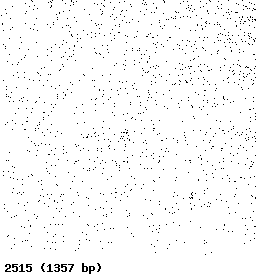

Supplement: Supplementary file 3 — Supplementary material [file mmc3.zip › CGR 2515.png]

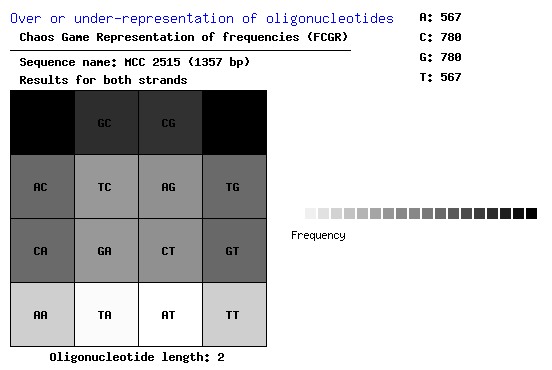

Supplement: Supplementary file 4 — Supplementary material [file mmc4.zip › FCGR 2515.png]

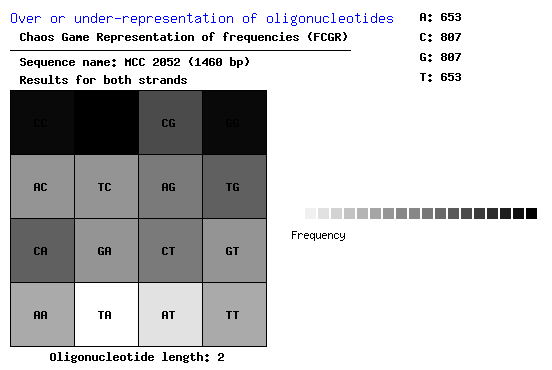

Supplement: Supplementary file 4 — Supplementary material [file mmc4.zip › FCGR 2052.png]

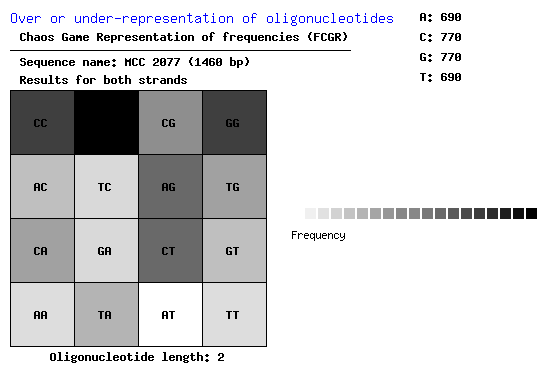

Supplement: Supplementary file 4 — Supplementary material [file mmc4.zip › FCGR 2077.png]

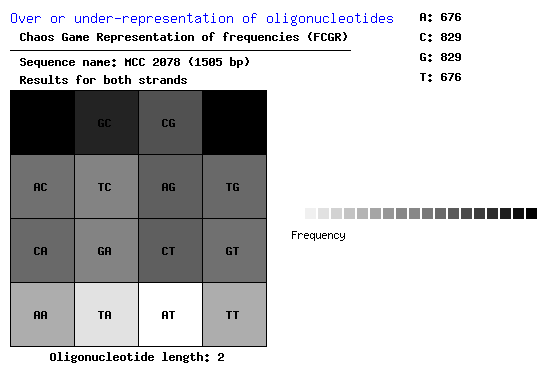

Supplement: Supplementary file 4 — Supplementary material [file mmc4.zip › FCGR 2078.png]

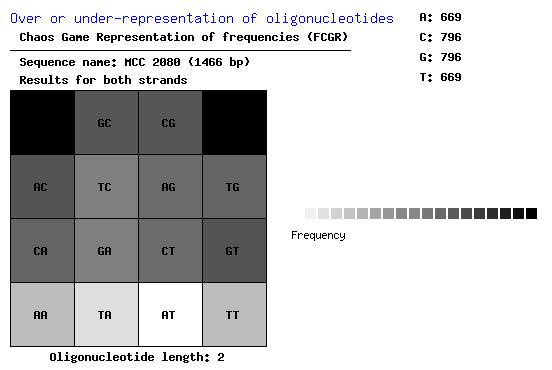

Supplement: Supplementary file 4 — Supplementary material [file mmc4.zip › FCGR 2080.png]

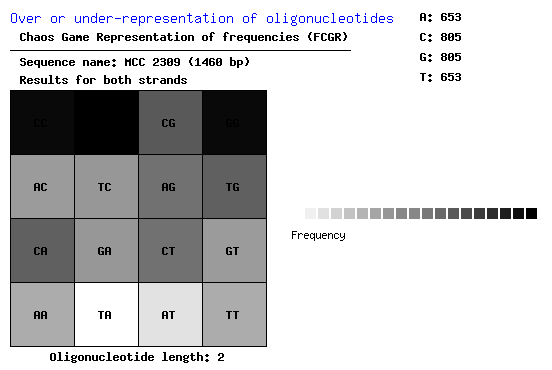

Supplement: Supplementary file 4 — Supplementary material [file mmc4.zip › FCGR 2309.png]

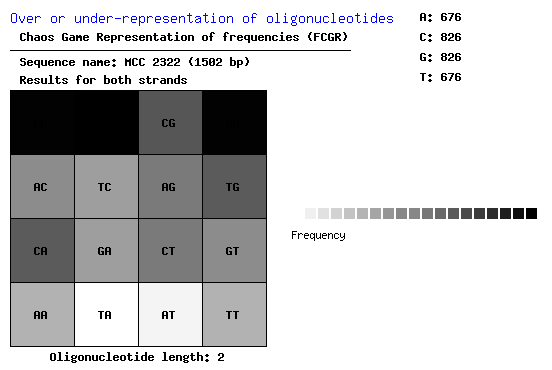

Supplement: Supplementary file 4 — Supplementary material [file mmc4.zip › FCGR 2322.png]

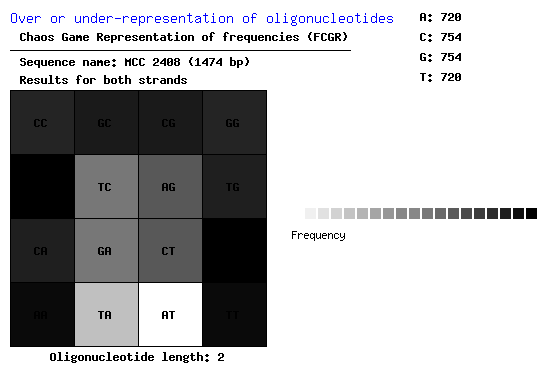

Supplement: Supplementary file 4 — Supplementary material [file mmc4.zip › FCGR 2408.png]

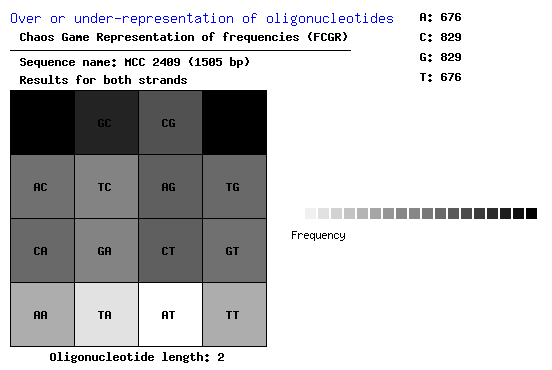

Supplement: Supplementary file 4 — Supplementary material [file mmc4.zip › FCGR 2409.png]

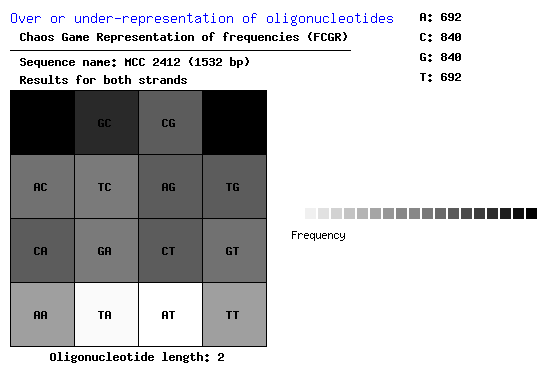

Supplement: Supplementary file 4 — Supplementary material [file mmc4.zip › FCGR 2412.png]

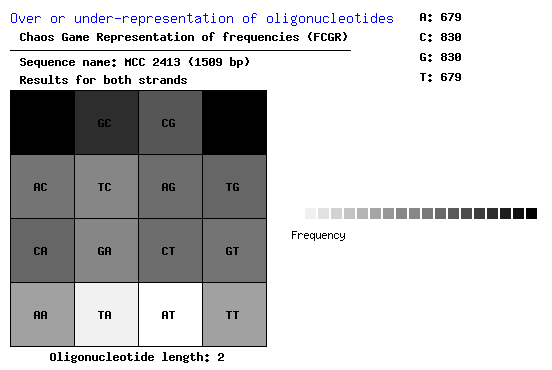

Supplement: Supplementary file 4 — Supplementary material [file mmc4.zip › FCGR 2413.png]

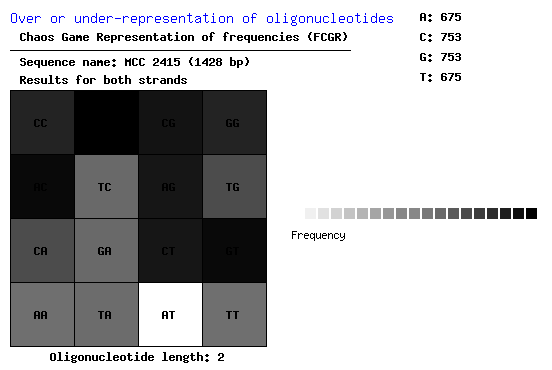

Supplement: Supplementary file 4 — Supplementary material [file mmc4.zip › FCGR 2415.png]

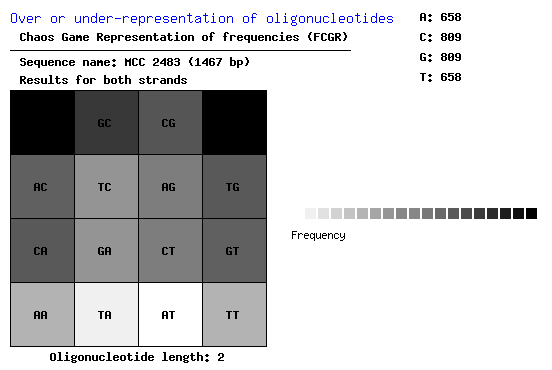

Supplement: Supplementary file 4 — Supplementary material [file mmc4.zip › FCGR 2483.png]

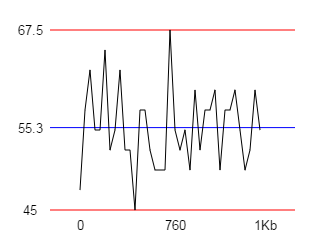

Supplement: Supplementary file 5 — Supplementary material [file mmc5.zip › GC CONTENT/2052.png]

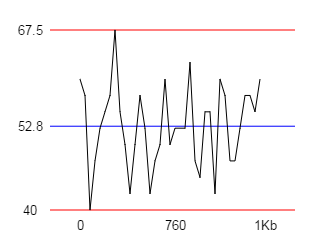

Supplement: Supplementary file 5 — Supplementary material [file mmc5.zip › GC CONTENT/2077.png]

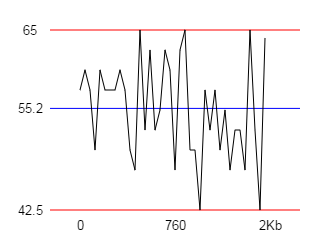

Supplement: Supplementary file 5 — Supplementary material [file mmc5.zip › GC CONTENT/2078.png]

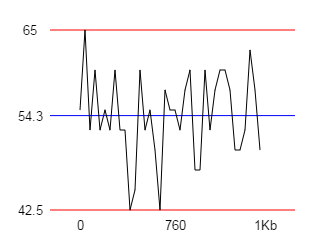

Supplement: Supplementary file 5 — Supplementary material [file mmc5.zip › GC CONTENT/2080.png]

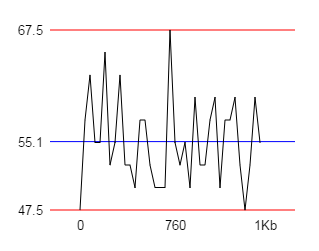

Supplement: Supplementary file 5 — Supplementary material [file mmc5.zip › GC CONTENT/2309.png]

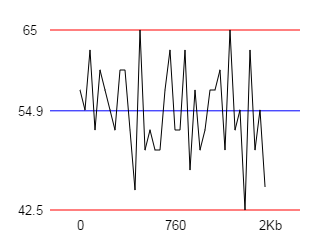

Supplement: Supplementary file 5 — Supplementary material [file mmc5.zip › GC CONTENT/2322.png]

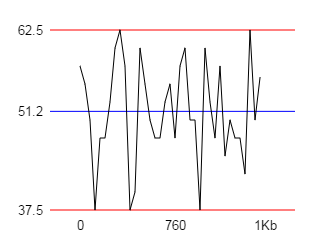

Supplement: Supplementary file 5 — Supplementary material [file mmc5.zip › GC CONTENT/2408.png]

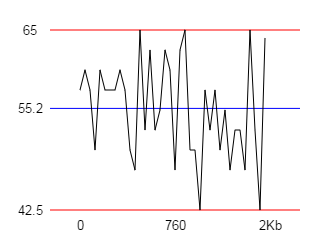

Supplement: Supplementary file 5 — Supplementary material [file mmc5.zip › GC CONTENT/2409.png]

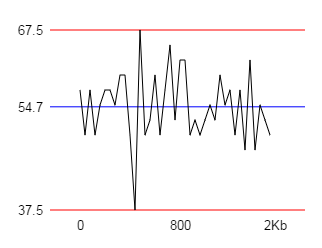

Supplement: Supplementary file 5 — Supplementary material [file mmc5.zip › GC CONTENT/2412.png]

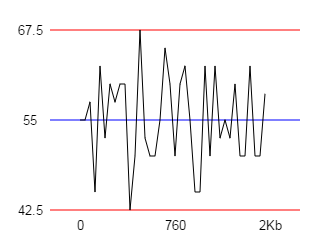

Supplement: Supplementary file 5 — Supplementary material [file mmc5.zip › GC CONTENT/2413.png]

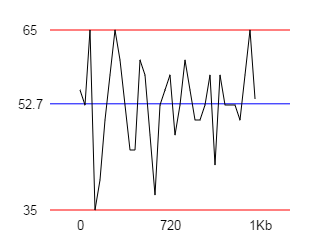

Supplement: Supplementary file 5 — Supplementary material [file mmc5.zip › GC CONTENT/2415.png]

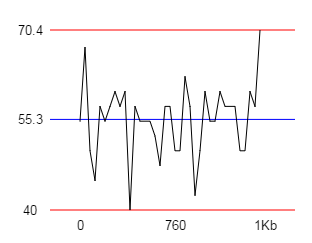

Supplement: Supplementary file 5 — Supplementary material [file mmc5.zip › GC CONTENT/2483.png]

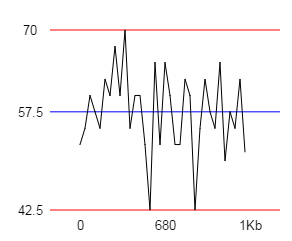

Supplement: Supplementary file 5 — Supplementary material [file mmc5.zip › GC CONTENT/2515.png]
